# Uptake of intermittent preventive treatment for malaria during pregnancy with Sulphadoxine-Pyrimethamine (IPTp-SP) among postpartum women in Zomba District, Malawi: a cross-sectional study

**DOI:** 10.1186/s12884-018-1744-y

**Published:** 2018-04-20

**Authors:** Steven Chifundo Azizi, Gershom Chongwe, Helen Chipukuma, Choolwe Jacobs, Jessy Zgambo, Charles Michelo

**Affiliations:** 1 Department of Epidemiology and Biostatistics, School of Public Health, University of Zambia, Post Box 50110, Lusaka, Zambia; 2 Malawi Defence Force, Malawi Military Health Services, Kamuzu Barracks, Private Bag 43, Lilongwe, Malawi; 3 Department of Health Policy and Management, School of Public Health, University of Zambia, Post Box 50110, Lusaka, Zambia

**Keywords:** Pregnancy, Intermittent preventive treatment, Sulphadoxine-pyrimethamine, Malaria, Uptake, Postpartum, Malawi

## Abstract

**Background:**

Malaria in pregnancy causes adverse birth outcomes. Intermittent preventive treatment of malaria during pregnancy with sulphadoxine-pyrimethamine (IPTp-SP) is recommended as a chemoprevention therapy. Zomba district IPTp uptake falls far below the national average. The study was conducted to assess determinants of IPTp-SP uptake during pregnancy among postpartum women in Zomba district after adoption of new IPTp-SP policy in 2014.

**Methods:**

This was a cross-sectional survey. Two public health facilities (HFs) were randomly selected from urban and rural areas in Zomba district. Study participants were postpartum women selected by using exit poll method from HFs. A total of 463 postpartum women were interviewed using structured questionnaire. Bivariate and multiple logistic regression was used in data analysis.

**Results:**

Out of all the enrolled participants (*n* = 463), 92% women had complete information for analysis. Of these, (*n* = 426) women, 127 (29.8%, 95% CI: 25.6%–34.3%) received three or more doses of SP, 299 (70.2%, 95% CI: 65.7%–74.4%) received two or less doses. Women receiving SP from rural HF were less likely to get at least three doses of SP than urban women, (AOR = 0.31, 95% CI 0.13–0.70); Others less likely were those with three or few antenatal care (ANC) visits versus four or more visits (AOR = 0.29, 95% CI 0.18–0.48); not taking SP under direct observation therapy (DOT) (AOR = 0.18, 95% CI (0.05–0.63).

**Conclusions:**

There is low utilisation of at least three doses of SP in this population and this seems to be associated with the number of ANC visits and use of DOTs. These determinants may therefore be important in shaping interventions aimed at increasing the uptake of IPTp in this district. In addition, the rural urban differential suggests the need for further research to understand the barriers and enablers of uptake in each context in order to improve the health of the community.

**Electronic supplementary material:**

The online version of this article (10.1186/s12884-018-1744-y) contains supplementary material, which is available to authorized users.

## Background

Malaria in pregnancy (MiP) is a significant cause of maternal morbidity and poor birth outcomes yet is preventable and treatable [1–4]. Women are particularly predisposed to adverse effects of malaria during their first and second pregnancies [5]. Globally, it is estimated that 125 million pregnant women are at risk of malaria infection each year and 30 million come from sub-Saharan Africa [6]. Furthermore, 75,000–200,000 infants and 10, 000 women deaths are estimated annually and the deaths are attributed to MiP [6, 7]. Particularly, *Plasmodium falciparum* infections in pregnancy contributes to approximately 11% (100, 000) of neonatal deaths due to low birth weight in areas of Africa where malaria is endemic (LBW) [7–9]. Infection with malaria in pregnancy predisposes women to increased risk of severe anaemia, intra-uterine growth retardation, intrauterine death, stillbirth, pre-term delivery, low birth weight (LBW), maternal death and placental malaria; and there is increased risk to unborn baby from miscarriage, stillbirth and LBW [10–12].

World Health Organisation (WHO) recommends three-pronged approach in stable malaria transmission areas for malaria prevention and control in pregnancy, which are Intermittent Preventive Treatment of malaria during pregnancy with sulphadoxine-pyrimethamine (IPTp-SP), use of insecticides treated nets (ITNs), and prompt diagnosis and effective treatment. In the IPTp-SP interventional regimen, each dose contains three tablets of sulphadoxine/pyrimethamine and each tablet contains 500 mg/25 mg SP [13, 14]. Although in 2004, WHO recommended a minimum of two doses of IPTp-SP during pregnancy [15, 16], in October 2012 this policy was updated to “at least three doses during pregnancy and to be administered at least 1 month apart at each scheduled antenatal care (ANC) visit, beginning from second trimester under direct observational therapy” [17, 18]. Malawi adopted the updated policy in 2013 [19]. WHO envisage the policy would increase IPTp-SP uptake as it also recommends at least four ANC visits during the second and third trimesters of pregnancy under the Focused antenatal care (FANC) model [17, 20]. Roll Back Malaria set a target of 100% utilization of at least two doses of IPTp-SP by 2015 [21]. Antenatal Care is the main point of contact of pregnant women with the health system [7]. ANC clinics are used as a vehicle of coverage of health interventions against malaria and other adverse conditions. Thus, the uptake of IPTp-SP would depend on attendance of pregnant women and delivery of the intervention at a health facility [16, 22].

Studies that estimated proportions of uptake of at least three doses of IPTp-SP and identified factors associated with it among pregnant women are few since WHO updated the IPTp-SP policy. However, previous studies revealed that, successful uptake of at least two doses of IPTp-SP is determined by the number of ANC visits [4, 18, 23–28], directly observed therapy (DOT) [4, 29, 30], residential area [18, 31], age of woman [31], education level and socioeconomic status [4, 32], parity [4, 31], timing of initial ANC visit [4, 33], knowledge about malaria/IPTp [4] and stockouts of the commodity [4]. This study envisages to contribute to identifying obstacles to completion of recommended doses of SP during pregnancy using the updated policy in order to develop a robust and multidisciplinary implementation approach for IPTp-SP intervention in resource limited settings. The aims of the study were to estimate the proportion of and identify factors determining the uptake of at least three doses of IPTp-SP among pregnant women in Zomba district, Malawi.

## Methods

### Study design and study settings

A cross sectional study was conducted in two health facilities in Zomba district, which is located in the southern region of Malawi between November 23, 2016 and January 27, 2017. Zomba district was purposively chosen because it was among the districts that had the lowest IPTp uptake in Malawi [34].

### Study population

The target population was all pregnant women and the study population was all postpartum women aged 15–49 who had just delivered (< 48 h) and those that delivered at home but visited the health facility for check-up at a randomly sampled Government-owned health facility in Zomba district.

### Inclusion and exclusion criteria

The study included postpartum women of the ages between 15 to 49 years who delivered at Government Health facility (HF), or delivered at home but brought a baby to HF within 48 h for check-up to minimise recall bias. They had singleton pregnancy, absence of reported antimalarial treatment other than SP in the previous 1 month. On the other hand, complicated cases of pregnancy, postpartum women on cotrimoxazole prophylaxis during their pregnancy because of increased risk of adverse drug reactions when taken together with SP were excluded from the study [14, 35].

### Variables

Guided by literature review, the study included the following variables that had been theoretically and empirically linked to malaria in pregnancy and uptake of IPTp-SP. The outcome was IPTp-SP uptake (≤2 doses versus 3+ doses) and. Explanatory variables were age, marital status, residence (location of the health facility), level of education, occupation, religion, tribe, knowledge about malaria transmission, knowledge about dangers of malaria in pregnancy, parity, number and timing of antenatal clinic visits, taking SP each time under directly observed therapy, distance to health facility, need for spouse’s escort to ANC, worried no health care provider and drugs at health facility and gravida (Table 1).

**Table 1:** Variables used in the study

Variable	Definition	Measurement scale
IPTp-SP uptake (Primary outcome)	Two or less (≤2) doses is incomplete and three or more (3+) is optimal	Nominal/binary
Explanatory variable		
Age group	Age of a woman	Nominal
Marital status	Marital status of a woman	Nominal
Residence (Zone)	Type of woman’s residence	Nominal/binary
Education	Level of education of woman	Nominal
Occupation	Woman’s occupation	Nominal
Religion	Woman’s religion	Nominal
Tribe	Woman’s tribe	Nominal
First pregnancy	Woman’s first pregnancy	Nominal/binary
Parity	Number of birth that a woman had after 20 weeks gestation	Nominal
Timing of 1st ANC visit	Age of the pregnancy a first time a woman visited antenatal care clinic (ANC)	Nominal
ANC visits	Number of antenatal care visits a pregnant woman made during her gestation period	Nominal
Gravida	Total number of confirmed pregnancies that a woman had regardless of the outcome	Nominal
Knowledge of malaria transmission	Three items (sugarcane, mosquito, witchcraft). If only mosquito is selected as the an agent of transmission, then the person is knowledgeable, otherwise inadequate knowledge	Nominal/binary
Knowledge of dangers of malaria in pregnancy	Three items (abortion, still birth, low birth weight) indicated knowledgeable of consequences of malaria in pregnancy. Otherwise, inadequate knowledge	Nominal/binary
Took SP under DOT each time	Whether a woman took SP under direct observation therapy (DOT) strategy or not	Nominal/binary
Permission to go to HF	Seeking permission from spouse to visit health facility (HF) for ANC	Nominal/binary
Distance to HF	Distance to health facility from a woman’s residence	Nominal/binary
Transport to HF	Transportation to heath facility	Nominal/binary
Need for spouse escort to HF	Spouse escort to health facility	Nominal/binary
Worried no health provider at HF	Worried that there would be no health care provider	Nominal/binary
Worried no drugs at HF	Worried that there might be no drugs at clinic	Nominal/binary

### Sampling and sample size determination

The district was divided into two sites according to residential settings: rural and urban. All Government owned health facilities that offered maternity and antenatal services were included in the stratified sampling frame. There were seven public health facilities out of 15 that were offering maternity and antenatal services in rural. Only two public health facilities among five offered maternity and antenatal services in urban. One health facility was selected from each stratum using simple random sampling drawn from Excel spreadsheet “RAND()” command.

Sample size was determined using a single population proportion estimation formula and calculated using Epi Info 7 with 43.5% proportion [34], 5% of decision precision, 95% confidence interval and non-response rate of 20%. The calculated overall sample size was 454.

Since the number of deliveries varies for different health facilities, a proportionate method was used for determining the sample size for each facility. The sample for each facility was calculated by weighting the total sample size required with the relative proportion of clients (total deliveries for each facility, as reported by the heads of the facilities) for the week prior to beginning of data collection as numerator; and the sum of deliveries in all the facilities as denominator [36]. Systematic sampling was employed to choose postpartum women as they existed from the labour ward at a specified interval by using the estimate of the average clinic deliveries to calculate sampling interval. The first participant was enrolled at random. The random selection was done by entering all the names of women discharged at a particular day in Excel spreadsheet and executed RAND() command.

### Data collection and quality management

A total of six public nurses were recruited and trained in data collection methods and management for 2 days, followed by one-day fieldwork. This was done to ensure the data collected were consistent across data collectors, complete and valid. The investigator monitored and supervised the data collectors throughout the data collection exercise.

The study used a structured interviewer-administered questionnaire with some questions adapted from 2014 Malawi Malaria Indicator Survey and 2010 Malawi Demographic and Health Survey [34, 37] (Additional file 1). The questionnaire solicited data on demographic, number of IPTp-SP doses a woman received during pregnancy, knowledge about malaria and IPTp-SP, number of and timing of antenatal clinic visits, parity and gravida. Number of ANC visits, number of IPTp-SP doses received, date of the first ANC visit, and woman’s age were the data that were obtained from the questionnaire and triangulated by women’s ANC cards and health facility records. The cards and records were used to verify verbal reports and whenever there was information discordance, the cards and records data were collected.

The questionnaire was administered by trained nurses (interviewers). The English version questionnaire was translated into Chichewa, which is a local language by a multilingual person. Another individual with knowledge of both English and Chichewa translated the Chichewa version back to English to check consistency in meaning. The questionnaire was pre-tested on 30 postpartum women from peri-urban health facility. Results from the pre-test exercise were discussed among the research team and a revised version of the questionnaire was adopted to be used in data collection. During data collection process, the investigator checked 15% of the completed questionnaires for completeness and consistency and where necessary provided feedbacks to data collectors.

A template of the questionnaire was prepared using EpiData 3.1 (CDC, Atlanta, GA, USA) and data were entered into the software and validated by a second data entry (double entry). The data were then exported into Stata version 14.2 (Stata Corp, College Station, Tx, USA) for further cleaning and analysis.

### Data analysis

Data were summarised in the form of proportions and frequencies for categorical variables. Means with their respective standard deviations were used to summarise the data if the variables were continuous and normally distributed, otherwise median and respective interquartile range were used.

Bivariate binary logistic regression analysis was performed to determine the presence or absence of association between outcome and respective explanatory variables by generating crude odds ratios (OR). All explanatory variables with *p* < 0.20 in the bivariate analysis were included in both automated and investigator-led backward-stepwise multiple binary logistic regression analysis to further examine the association between the outcome and each explanatory variable while controlling the effects of other explanatory variables by calculating adjusted odds ratios (AOR) The level of significance used was 5% (0.05), two-tailed at 95% confidence interval (CI). The CI for proportions were calculated using logit transformation, which is a default option in Stata.

### Assessment of goodness of fit of the models

The goodness of fit of the models was tested using likelihood ratio (LR), and Homsmer and Lemeshow tests. The full model consisting of all the independent variables was compared with the model created by backward stepwise regression at 5% significant level. Test result with significant level above 5% was interpreted as a model with fewer independent variables and better than the full model. The classification ability of the models was evaluated using Receiver Operating Characteristics (ROC) curve. The ROC value above 0.5 meant that a model classification was not due to chance.

**Fig. 1:**
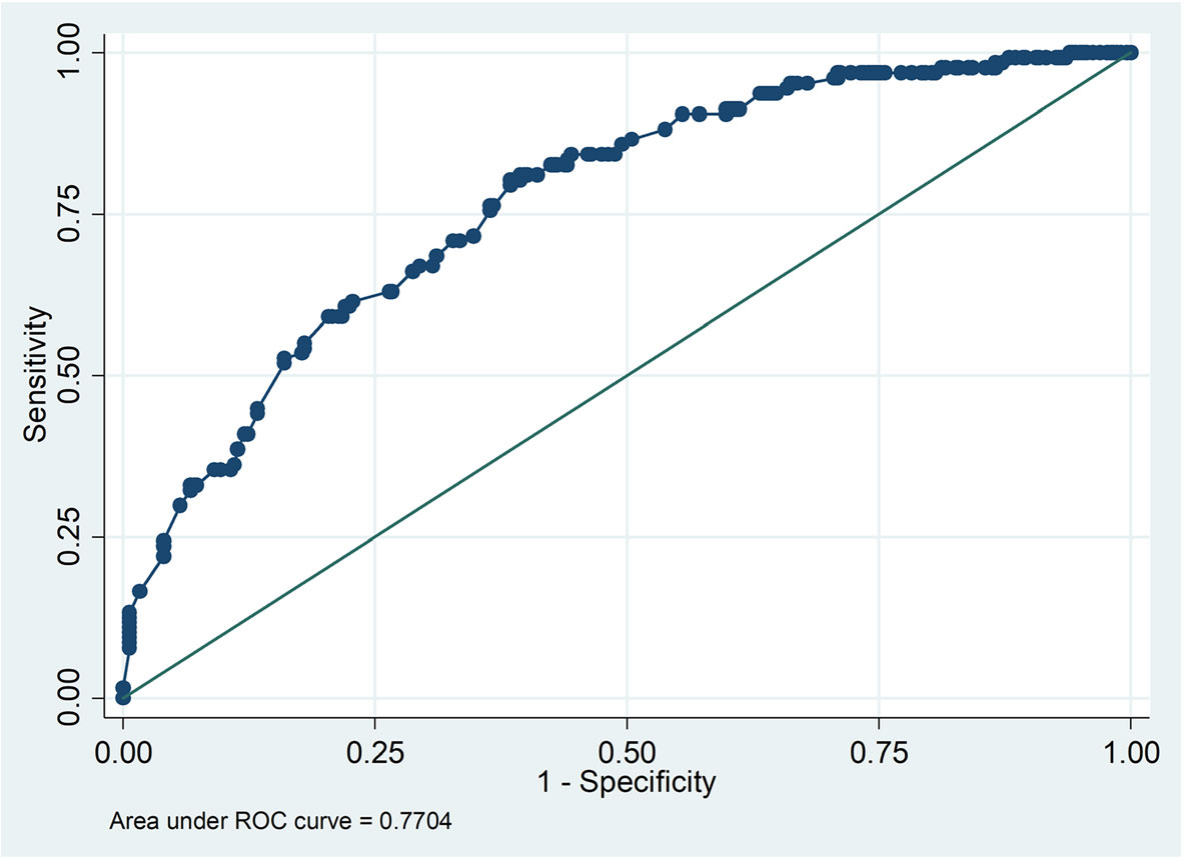
Receiver Operating Characteristic (ROC) curve

The Likelihood ratio (LR) chi-square with 22° degrees of freedom was 14.09, *p*-value = 0.898. This means the model with fewer (nine) explanatory variables was better model than the one with 21 variables. The Hosmer-Lemeshow goodness-of-fit test was found to be not significant (χ^2^ = 8.39, *df* = 8, *p* = 0.396). Thus we do not have enough evidence to reject the null hypothesis that the model fitted the data well. Furthermore, ROC value for the selected model as shown in Fig. 1 is 0.77, which suggests that classification is not due to chance as ROC value is close to 1 than 0.5.

### Ethical approval and consent to participate

Ethics approval was obtained from National Health Sciences Research Committee in Malawi (issued September 12, 2016, protocol number 1656) and University of Zambia Biomedical Research Ethics Committee in Zambia (issued August 3, 2016, reference number 017–06-16). Written permission to conduct the study in the health facilities (Matawale and Likangala) was obtained from Zomba District Health Office health facilities involved in the study. Informed written consents and assents were obtained from study participants whose age was 18 years and above and below 18 years respectively. Willingness to participate in the study and parental permission was confirmed by signing (finger print for illiterates) on the informed consent sheet.

## Results

### Prevalence of IPTP-SP uptake

A total of 463 women were enrolled into the study. Out of this, 426 (92%) women with complete information had their data analysed to investigate the determinants of IPTp-SP uptake. Of 426 women, 127 (29.8%, 95% CI: 25.6%–34.3%) received three (optimal) or more doses of SP, 299 (70.2%, 95% CI: 65.7%–74.4%) received two or less doses (poor uptake or suboptimal).

### Sociodemographic characteristics of participants

Out of 426 women, 55% delivered at urban health facility and 45% delivered at the rural health facility. Of 233 women who delivered at urban health facility, 40% took three or more doses and 60% received two or less doses of SP during pregnancy. Among 193 delivering women at rural health centre, 17% received three or more doses while 83% took two or less doses of SP. More than half (232, 54%) of participants had completed at least senior primary school. Pregnant women in the age group 25–35 had the highest uptake of optimal SP doses compared to the rest of the women. Women who were divorced or separated or widowed had highest percentage of taking optimal SP doses (Table 2).

**Table 2:** Sociodemographic characteristics of participants by IPTp uptake

Characteristic	*N* (Total = 426)	% of women who took IPTp			
		≤2 doses^*a*^	95% CI	3+ doses^*b*^	95% CI
		n (%)		n (%)	
Zone					
Urban	233	139 (59.7)	53.2–65.8	94 (40.3)	34.2–46.8
Rural	193	160 (82.9)	76.9–87.6	33 (17.1)	12.4–23.1
Education					
No formal education/junior primary	194	130 (67.0)	60.0–73.3	64 (33.0)	26.7–39.9
Senior primary	125	97 (77.6)	69.4–84.1	28 (22.4)	15.9–30.
Secondary/tertiary	107	72 (67.3)	57.8–75.6	35 (32.7)	24.4–42.2
Age group					
15–24	201	146 (72.6)	66.0–78.4	55 (27.4)	21.6–33.9
25–34	176	114 (64.8)	57.4–71.5	62 (35.2)	28.5–42.6
35+	49	39 (79.6)	65.9–88.7	10 (20.4)	11.3–34.1
Occupation					
Unemployed	307	228 (74.3)	69.1–78.9	79 (25.7)	21.1–30.9
Self-employed	104	64 (61.5)	51.8–70.4	40 (38.5)	29.6–48.2
Employed	15	7 (46.7)	23.4–71.5	8 (53.3)	28.5–76.6
Marital status					
Married	382	272 (71.2)	66.4–75.5	110 (28.8)	24.5–33.6
Divorced/separated/widowed	19	10 (52.6)	30.5–73.8	9 (47.4)	26.2–69.5
Never married	25	17 (68.0)	47.3–83.4	8 (32.0)	16.6–52.7
Religion					
CCAP^*c*^/7th day^*d*^/Baptist	112	70 (62.5)	53.1–71.0	42 (37.5)	28.9–46.9
Other Christian	134	102 (76.1)	68.1–82.6	32 (23.9)	17.4–31.9
Catholic/Anglican	93	65 (69.9)	59.8–78.4	28 (30.1)	21.6–40.2
Muslim/other religions	87	62 (71.3)	60.8–79.8	25 (28.7)	20.2–39.2
Tribe					
Nyanja	106	85 (80.2)	71.4–86.8	21 (19.8)	13.2–28.6
Chewa	107	69 (64.5)	54.9–73.0	38 (35.5)	26.9–45.1
Lomwe	102	75 (73.5)	64.1–81.2	27 (26.5)	18.8–35.9
Other	111	70 (63.1)	53.7–71.6	41 (36.9)	28.4–46.3

^*a*^uptake of two or less doses of IPTp-SP

^*b*^uptake of three or more doses of IPTp-SP

^*c*^Church of Central Africa Presbyterian

^*d*^Seventh Day Adventist

### Cultural characteristics

Women who had problems with spouses’ escort to ANC clinic had higher percentage (42%) of receiving three or more doses than those who had no problem with escorts (23%). Participants with a small problem in getting permission from their spouse to visit HF had a slight higher percentage (30%) of completing SP doses than women who had a big problem with seeking permission (25%). Women who first visited ANC in first trimester had the highest percentage of completing the recommended doses (Table 3).

**Table 3:** Cultural characteristics by IPTp uptake

Characteristic	*N* (Total = 426)	% of women who took IPTp			
		≤2 doses	95% CI	3+ doses	95% CI
Permission to go to HF^*a*^					
Small problem	410	287 (70.0)	65.3–74.3	123 (30.0)	25.7–34.6
Big problem	16	12 (75.0)	48.2–90.6	4 (25.0)	9.4–51.8
Need for spouse escort to HF					
Small problem	276	212 (76.8)	71.4–81.4	64 (23.2)	18.6–28.6
Big problem	150	87 (58)	49.9–65.7	63 (42.0)	34.3–50.1
Timing of 1st ANC visit					
2nd trimester	284	198 (69.7)	64.1–74.8	86 (30.3)	25.2–35.9
1st trimester	92	56 (60.9)	50.5–70.4	36 (39.1)	29.6–49.5
3rd trimester	50	45 (90.0)	77.9–95.8	5 (10.0)	4.2–22.1

^*a*^Heath Facility

### Individual characteristics

The percentages for completing SP doses were similar for both primigravida and multigravida (29.9% vs 29.5% respectively). At least four ANC visits a pregnant woman made was associated with higher proportion of taking three or more SP doses than three or less visits. Both groups of women who were knowledgeable and partially knowledgeable about malaria transmission had similar percentages of taking three or more doses of SP (29.8% vs 30%). However, those women who were aware of dangers of malaria in pregnancy had higher percentage (32%) of completing recommended SP doses than those with inadequate knowledge (25%) (Table 4).

**Table 4:** **C**linical characteristics and level of knowledge among participants by IPTp uptake

Characteristic	N (Total = 426)	% of women who took IPTp			
		≤2 doses	95%CI	3+ doses	95% CI
		n (%)		n (%)	
First pregnancy					
No	304	213 (70.1)	64.7–75.0	91 (29.9)	25.0–35.3
Yes	122	86 (70.5)	61.8–77.9	36 (29.5)	22.1–38.2
Parity					
One child	121	85 (70.3)	61.5–77.8	36 (29.8)	22.2–38.5
Two children	111	72 (64.9)	55.5–73.2	39 (35.1)	26.8–44.5
3+ children	194	142 (73.2)	66.5–78.9	52 (26.8)	21.0–33.5
ANC visits					
4+	234	140 (59.8)	53.3–65.9	94 (40.2)	34.1–46.6
Three or less	192	159 (82.8)	76.8–87.5	33 (17.2)	12.5–23.2
Gravida					
multigravida^a^	194	141 (72.7)	65.9–78.5	53 (27.3)	21.5–34.1
Secundigravida^b^	110	72 (65.4)	56.0–73.8	38 (34.6)	26.2–43.9
primigravida^c^	122	86 (70.5)	61.8–77.9	36 (29.5)	22.1–38.2
Knowledge of malaria transmission					
knowledgeable	386	271 (70.2)	65.4–74.6	115 (29.8)	25.4–34.6
Inadequate Knowledge	40	28 (70.0)	54.0–82.3	12 (30.0)	17.7–46.0
Knowledge of dangers of malaria in pregnancy					
Knowledgeable	303	207 (68.3)	62.8–73.3	96 (31.7)	26.7–37.2
Inadequate knowledge	123	92 (74.8)	66.3–81.7	31 (25.2)	18.3–33.7

^a^a woman that has been pregnant for at least a second time

^b^a woman in her second pregnancy

^c^a woman who is pregnant for the first time

### Structural factors (access to health facility and provider readiness) faced by participant

Pregnant women who took SP under Direct Observation Therapy (DOT) each time had higher percentage of completing three or more doses than those who did not take SP under DOT each time they visited ANC (33% vs 6%). Participants who had problems with long distance and lack of transport to health facility had lower percentage of completing the recommended doses. The women who worried most that there would be no health provider at HF had lower percentage (20%) of completing the recommended doses against 35% for participants who felt otherwise (Table 5).

**Table 5:** IPTp uptake against factors that affect access to health facility and health provider DOT adherence

Characteristic	*N* (Total = 426)	% of women who took IPTp			
		≤2 doses	95%CI	3+ doses	95% CI
Took SP under DOT^a^ each time					
Yes	376	252 (67.0)	62.1–71.6	124 (32.9)	28.4–37.9
No	50	47 (94.0)	82.8–98.1	3 (6.0)	1.9–17.2
Distance to HF					
Small problem	348	233 (67.0)	61.8–71.7	115 (33.1)	28.3–38.2
Big problem	78	66 (84.6)	74.7–91.1	12 (15.4)	8.9–25.3
Transport to HF					
Small problem	344	231 (67.2)	61.9–71.9	113 (32.9)	28.1–38.0
Big problem	82	68 (82.9)	73.1–89.7	14 (17.1)	10.3–26.9
Worried no health provider at HF					
Small problem	280	183 (65.4)	59.6–70.7	97 (34.6)	29.3–40.4
Big problem	146	116 (79.5)	72.1–85.3	30 (20.0)	14.7–27.9
Worried no drugs at HF					
Small problem	263	172 (65.4)	59.4–70.9	91 (34.6)	29.1–40.6
Big problem	163	127 (77.9)	70.8–83.6	36 (22.1)	16.3–29.1

^a^Direct Observation Therapy

### Determinants of uptake of IPTp-SP

#### Bivariate analysis

Bivariate logistic regression results showed that 12 out of 21 predictor variables were significantly associated with completion of the recommended doses of SP during pregnancy (Table 6). Significant barriers to completion of recommended SP doses by a pregnant women: Attending ANC from health facility in rural setting compared to urban setting (OR = 0.30, 95% CI: 0.19–0.48, *p* < 0.001), women who attained senior primary school education than women without formal or junior primary education (OR = 0.58, 95% CI: 0.35–0.98, *p* = 0.043), being a member of other Christian denomination than a member of Church of Central Africa Presbyterian (CCAP) or Seventh Day Adventist or Baptist (OR = 0.52, 95% CI: 0.30–0.91, *p* = 0.021), commencing ANC in third trimester than in second trimester (OR = 0.26, 95% CI: 0.09–0.67, *p* = 0.005), three or less number of ANC visits against four or more visits (OR = 0.31, 95% CI: 0.19–0.49, *p* < 0.001), not taking SP doses under direct observation of health care provider (OR = 0.13, 95% CI: 0.04–0.43, *p* = 0.001), distance to health facility (OR = 0.37, 95% CI: 0.19–0.71, *p* = 0.003), transport problem to health facility (OR = 0.42, 95% CI: 0.23–0.78, *p* = 0.006), and worried that there would be no drugs at health facility (OR = 0.54, 95% CI: 0.34–0.84, *p* = 0.006).

**Table 6:** Factors associated with uptake of three or more doses of IPTp-SP among postpartum women, *N* = 426

Characteristics	N	Crude Odds Ratio (95%CI)	Unadjusted *p*-value^*a*^	Adjusted Odds Ratio (95%CI)	Adjusted *p*-value^*b*^
Zone					
Urban	233	1		1	
Rural	193	0.30 (0.19–0.48)	< 0.001	0.31 (0.13–0.70)	0.005
Education					
No formal education/junior primary	194	1			
Senior primary	125	0.58 (0.35–0.98)	0.043		
Secondary/tertiary	107	0.99(0.60–1.63)	0.961		
Age group					
15–24	201	1		1	
25–34	176	1.44 (0.93–2.23)	0.100	1.72 (1.06–2.78)	0.028
35+	49	0.68 (0.32–1.46)	0.322		
Occupation					
Unemployed	307	1			
Self-employed	104	1.80 (1.13–2.89)	0.014		
Employed	15	3.30 (1.15–9.39)	0.025		
Marital status					
Married	382	1		1	
Divorced/separated/widowed	19	2.23 (0.88–5.63)	0.091	2.58 (0.90–7.39)	0.078
Never married	25	1.16 (0.49–2.77)	0.733		
Religion					
CCAP/7th day/Baptist	112	1		1	
Other Christian	134	0.52 (0.30–0.91)	0.021	0.61 (0.35–1.05)	0.075
Catholic/Anglican	93	0.72 (0.40–1.29)	0.267	0.53 (0.28–0.97)	0.041
Muslim/other religions	87	0.67 (0.37–1.23)	0.195		
Tribe					
Nyanja	106	1			
Chewa	107	2.23 (1.19–4.15)	0.011		
Lomwe	102	1.46 (0.76–2.79)	0.256		
Other	111	2.37 (1.28–4.38)	0.006		
First pregnancy					
No	304	1			
Yes	122	1.04 (0.65–1.64)	0.883		
Parity					
One child	121	1			
Two children	111	1.28 (0.74–2.23)	0.382		
3+ children	194	0.86 (0.52–1.43)	0.571		
Timing of 1st ANC visit					
2nd trimester	284	1			
1st trimester	92	1.48 (0.91–2.41)	0.116		
3rd trimester	50	0.26 (0.09–0.67)	0.005		
ANC visits					
4+	234	1		1	
Three or less	192	0.31 (0.19–0.49)	< 0.001	0.29 (0.18–0.48)	< 0.001
Gravida					
multigravida	194	1			
Secundigravida	110	1.40 (0.85–2.32)	0.187		
primigravida	122	1.11 (0.67–1.84)	0.674		
Knowledge of malaria transmission					
knowledgeable	386	1			
Inadequate Knowledge	40	1.01 (0.50–2.06)	0.978		
Knowledge of dangers of malaria in pregnancy					
Knowledgeable	303	1			
Inadequate knowledge	123	0.73 (0.45–1.17)	0.186		
Took SP under DOT each time					
Yes	376	1		1	
No	50	0.13 (0.04–0.43)	0.001	0.18 (0.05–0.63)	0.007
Permission to go to HF					
Small problem	410	1			
Big problem	16	0.78 (0.25–2.46)	0.669		
Distance to HF					
Small problem	348	1		1	
Big problem	78	0.37 (0.19–0.71	0.003	0.49 (0.23–1.06)	0.070
Transport to HF					
Small problem	344	1			
Big problem	82	0.42 (0.23–0.78)	0.006		
Need for spouse escort to HF					
Small problem	276	1		1	
Big problem	150	2.40 (1.56–3.68)	< 0.001	2.03 (1.26–3.26)	0.004
Worried no health provider at HF					
Small problem	280	1			
Big problem	146	0.49 (0.30–0.78)	0.003		
Worried no drugs at HF					
Small problem	263	1		1	
Big problem	163	0.54 (0.34–0.84)	0.006	1.84 (0.82–4.12)	0.140

^*a*^Bivariate logistic regression

^*b*^Multiple logistic regression

The enabling determinants significantly associated with a pregnant woman to more likely to complete recommended doses were: being self-employed woman versus unemployed women (OR = 1.80, 95% CI: 1.13–2.89, *p* = 0.014), being employed against unemployed (OR = 3.30, 95% CI: 1.15–9.39, *p* = 0.025), being Chewa tribe than Nyanja tribe (OR = 2.23, 95% CI: 1.19–4.15, *p* = 0.011), being of other tribe rather than Nyanja (OR = 2.37, 95% CI: 1.28–4.38, *p* = 0.006), and having problems with spouse escort to health facility (OR = 2.40, 95% CI: 1.56–3.68, *p* < 0.001).

#### Multivariable analysis

In multiple binary logistic regression results, six out of nine explanatory variables were significantly associated with a pregnant woman receiving at least three doses of SP. Only two enablers were significantly associated with a pregnant woman completing at least recommended SP doses. First, being in the age group 25–34 compared to age group 15–24 (AOR = 1.72, 95% CI: 1.06–2.78, *p* = 0.028). Second, pregnant women with problem of spouse escort to health facility (AOR = 2.03, 95% CI: 1.26–3.26, *p* = 0.004).

The following determinants made a pregnant woman less likely to complete at least the recommended doses of SP after adjusting for other independent variables. Pregnant women who attended ANC from health facility in rural setting than those from urban settings (AOR = 0.31, 95% CI: 0.13–0.70, *p* = 0.005), pregnant women who had three or less ANC visits (AOR = 0.29, 95% CI: 0.18–0.48, *p* < 0.001), women who did not take doses of SP under direct observations of health care provider (AOR = 0.18, 95% CI: 0.05–0.63, *p* = 0.007), and being a Catholic or Anglican member compared to a member of Church of Central Africa Presbyterian (CCAP) or Seventh Day Adventist (AOR = 0.53, 95% CI: 0.28–0.97, *p* = 0.041). The results are presented in Table 6.

## Discussion

The WHO new recommended doses of IPTp-SP uptake was very low in the study population when comparing to Roll Back Malaria (RBM) benchmark target for all pregnant women in areas with moderate-to-high transmission in Africa [7]. Some of the factors associated with the low uptake that the study found were attending ANC clinic in rural area, three or less ANC visits, not taking SP under DOT.

Health facility location/women’s residential area setting has been found in this study to be related to uptake of IPTp-SP. Women who attended rural health facility were less likely to complete the recommended SP doses during pregnancy. This finding is similar to a study done in Geita district, Tanzania [18] and Nigeria [36]. To the best knowledge of the authors, the Geita-Tanzania study was the only study that evaluated uptake of IPTp-SP, categorised into less than three doses versus three or more doses of SP, at the time of writing this paper. This observation might either indicate that rural health facility had lower stock levels of SP than urban health facility because of poor supply chain from district health office (DHO) due to challenges in transportation of the commodity or understaffing [4] which leads to high client-to-staff ratios and subsequently long queues and waiting times. This could prompt some pregnant women not make further ANC visits. Kibusi et al. [31] reported that being a resident of Eastern Zone (urban) in Tanzania was associated with completing two or more doses of SP. This implies that Zomba District Health Office (ZDHO) should strive to strength the rural health systems and achieve equity. On the other hand, there is need for further investigation into the rural-urban differential to understand the real barriers and enablers of uptake in each context in order to improve the health of the community.

This study has shown that receipt of at least three doses of SP was higher among pregnant women making four or more ANC clinic visits than those making three or less visits. The finding is consistent with a study conducted in Tanzania [18, 24], in Ghana [27], in Burkina Faso [25], and in Mali [26, 28] in Cameroon, and in Benin [23]. ANC clinic is the vehicle that carries the intervention from the healthcare provider to the pregnant woman, hence the more visits a pregnant woman makes to ANC clinics the higher the number of SP doses she would receive as long as the visits are scheduled at least 1 month apart beginning from second trimester. Out of 426 women, 234 (55%) made at least four ANC visits but only 94 out of 234 (40%) received optimal doses of SP. This suggests a missed opportunity to provide the recommended doses of SP to women who attended WHO recommended number of at least four scheduled ANC visits, as two-thirds (284 out of 426) of the women commenced ANC clinic in the second trimester. The mismatch between percentage of four or more ANC visits and percentage of receipt of three or more doses could occur in part because of intermittent shortage of SP in the health facility, poor fidelity in implementation of IPTp-SP by individual healthcare providers as recommended in WHO policy brief [38], and women’s negative attitudes towards the use of the drug during pregnancy [4, 18, 26, 39]. This implies that health care providers and community leaders have to collaborate to aggressively promote optimal ANC attendance among pregnant women and make sure that every eligible pregnant woman who attends ANC at scheduled visit receives a dose of SP. This will maximise the opportunity of optimal SP uptake in the district for those who make four or more ANC visits.

In regards to direct observation therapy (DOT), the study has revealed that pregnant women who did not take doses of SP under DOT had less likelihood of completing the recommended number of doses. GM Mubyazi, P Magnussen, C Goodman, IC Bygbjerg, AY Kitua, OE Olsen, J Byskov, KS Hansen and P Bloch [40] observed that uptake of SP was low among the study participants especially when women were allowed to take the SP at home. There are many reasons why healthcare providers allowed women to take the drug home such as shortages of clean water and cups [40], high client-to-staff ratios which reduces consultation times resulting in poor or no observation [4, 30]. Taking doses of SP under direct observation therapy (DOT) of healthcare provider is one of the strategies in implementation of IPTp-SP with high fidelity [13]. This suggests that health care providers should strive to follow protocols in SP provision. Besides, health facilities’ management has to strengthen their efforts in supervising ANC providers and learn about challenges they face in order find solutions of strengthening DOT, which would work in their context. Considering that resources, such as cups and clean drinking water, will never be sufficient, ZDHO and health facility management ought to be pragmatic in overcoming this challenge. One solution might be engaging community leaders to encourage pregnant women to bring with them a cup when on a scheduled ANC visit.

The odds of completing at least three doses of SP was more than 1.72 times higher among pregnant women between age group 25 to 34 than those aged between 15 to 24 years old. This result indicates that young pregnant women were under utilizing the intervention, which is similar result that Kibusi’s study revealed [31]. Therefore, there is need to increase awareness of importance of completing recommended doses of IPTp-SP among pregnant women aged between 15 to 24 years as well pregnant women older than 34 years. The study has shown that poor uptake is affected by differentially on women born at different times, which might reflect women’s culture of health care utilization at different ages. For instant, teen pregnancies are usually unplanned therefore are less likely to consistently utilize ANC services and subsequently miss out on receiving optimal doses of SP.

Lastly, spouse escort to health facility has shown to be positively associated with good uptake of several interventions offered at ANC [41]. However, pregnant women in the study population who had a problem with spouse escort were more likely to complete the recommended doses of SP. Mostly, these women were either divorced or windowed or on separation, hence they were socially vulnerable and probably took their situation positively by attending ANC clinics and took the recommended SP doses to avoid MiP and its adverse effects. The other possible explanation would be that they had more autonomy in relation to the use of ANC than married women [42]. This might imply that pregnant women with more autonomy are in a position to have confidence in prioritization of health care services.

This study had the following limitations. First, the study participants were recruited from health facility maternity wings and antenatal clinics. In Malawi, according to National Statistical Office (NSO) [Malawi] and ICF Macro [34], 20% of births occur at home and 80% of women who received no antenatal care services deliver at home. Therefore, it would not be representative of the wider population of postpartum women in Zomba district. However, the researcher minimised the limitation by including women who delivered at home and visited the health facility within 48 h for check-up. Only 14% of women age 15–49 giving birth outside health facility received a postnatal check in the first 2 days after delivery [43]. Second, the health systems factors (time and patient flow challenges, integration of services into ANC); Policy and guidance environment, human resource factors, and supply chain factors (SP availability at the facility) also play an important role in the coverage of the intervention. However, this study did not study them because the focus was primarily on pregnant women’s perceptive. This limitation would under-or-overestimate the problem understudy when viewed holistically. Third, the study focused on determinants of IPTp-SP uptake in postpartum women from catchment areas of selected Health facilities in Zomba district. Hence, the results would not be generalised to all districts in Malawi because the participating district was purposively sampled because of its low uptake. However, the results could be related to other districts with similar characteristics as the sampled one. Finally, recall bias is an inherent limitation of the survey design. There was a possibility of women reporting past exposures/experiences with varying degree of accuracy. However, some of these recall biases were minimised by using records such as ANC cards and interviewing the women immediately after delivering.

## Conclusion

Malaria in pregnancy continues to pose a major public health problem in sub-Saharan Africa and particularly in Malawi. However, there are a number of evidenced-based interventions for preventing MiP and one of them is IPTp-SP. This study was conducted to identify the enablers and barriers of a pregnant woman completing at least three doses of IPTp-SP and estimate the uptake levels in Zomba district. The study demonstrated that there is low utilisation of SP in this population and this seems to be associated with the number of ANC visits, use of DOTs and health facility location. Therefore, community-based health promotion on optimal ANC attendance should be encouraged and antenatal care clinics should strengthen IPTp messages particularly on younger pregnant women. Zomba District Health Office in collaboration with health facilities’ management have to strengthen their efforts in supervising ANC providers to enhance DOT. Further research needs to be carry out to understand factors affecting the rural-urban differential IPTp uptake to provide evidence that would address the variance.

## Additional file

Additional file1: Woman questionnaire. The data collection tool that was administered to postpartum women. (DOC 325 kb)

## Abbreviations

ANC: Antenatal care
FANC: Focused antenatal care
IPTp1: Single dose of IPTp-SP
IPTp2: Two doses of IPTp-SP
IPTp3 +: Three or more doses of IPTp-SP
IPTp-SP: Intermittent preventive treatment in pregnacy with Sulphadoxine Pyrimethamine
ITNs: Insecticides treated nets
MiP: Malaria in pregnancy
SP: Sulphadoxine Pyrimethamine
WHO: World Health Organization
