# Supplementary material for: Uptake of intermittent preventive treatment for malaria during pregnancy with Sulphadoxine-Pyrimethamine (IPTp-SP) among postpartum women in Zomba District, Malawi: a cross-sectional study
Source: BMC Pregnancy Childbirth. 2018 Apr 20;18:108. doi: 10.1186/s12884-018-1744-y (PMC5910602; doi:10.1186/s12884-018-1744-y)
Supplement: Supplementary file 1 — Woman questionnaire. The data collection tool that was administered to postpartum women. (DOC 325 kb) [file 12884_2018_1744_MOESM1_ESM.doc]

**Woman Questionnaire**

| **Survey Information** | | | | | | | | | | | | | | | | | | | | | | | | |
| --- | --- | --- | --- | --- | --- | --- | --- | --- | --- | --- | --- | --- | --- | --- | --- | --- | --- | --- | --- | --- | --- | --- | --- | --- |
|  | | | **Response** | | | | | | | | | | | | | | | | | | | **Code** | | |
| Participant Identification number | | | **└─┴─┴─┴─┘** | | | | | | | | | | | | | | | | | | |  | | |
| Health Facility name | | |  | | | | | | | | | | | | | | | | | | |  | | |
| Zone (Residential Area) | | | urban | | | | | | 0 | | | | | | | | | | | | |  | | |
|  |  |  | rural | | | | | | 1 | | | | | | | | | | | | |  | | |
| Village name of Participant | | |  | | | | | | | | | | | | | | | | | | |  | | |
| Interviewer name | | |  | | | | | | | | | | | | | | | | | | |  | | |
| Signature of Interviewer | | |  | | | | | | | | | | | | | | | | | | |  | | |
| Date of interview | | | └─┴─┘ └─┴─┘ └─┴─┴─┴─┘  dd mm year | | | | | | | | | | | | | | | | | | |  | | |
| Duration of interview | | Time started  (24 hour clock) | └─┴─┘: └─┴─┘  hrs mins | | | | | | | | | | | | | | | | | | |  | | |
|  |  | Time ended  (24 hour clock) | └─┴─┘: └─┴─┘  hrs mins | | | | | | | | | | | | | | | | | | |  | | |
| **Consent, Interview Language and Name** | | | | | **Response** | | | | | | | | | | | | | | | | | | **Code** | |
| Consent has been read and obtained | | | | | Yes | | | | | | 1 | | |  | | | | | | | | |  | |
|  |  |  |  |  | No | | | | | | 0 | | | **If No, End** | | | | | | | | |  |  |
| Interview Language | | | | | Chichewa | | | | | | 1 | | |  | | | | | | | | |  | |
|  |  |  |  |  | Yao | | | | | | 2 | | |  | | | | | | | | |  |  |
|  |  |  |  |  | Lomwe | | | | | | 3 | | |  | | | | | | | | |  |  |
|  |  |  |  |  | Tumbuka | | | | | | 4 | | |  | | | | | | | | |  |  |
|  |  |  |  |  | English | | | | | | 5 | | |  | | | | | | | | |  |  |
|  |  |  |  |  | Other (Specify) | | | | | | 6 | | |  | | | | | | | | |  |  |
| Interview Result | | | | | Complete | | | | | | 1 | | |  | | | | | | | | |  | |
|  |  |  |  |  | Postponed | | | | | | 2 | | |  | | | | | | | | |  |  |
|  |  |  |  |  | Refused | | | | | | 3 | | |  | | | | | | | | |  |  |
|  |  |  |  |  | Partly completed | | | | | | 4 | | |  | | | | | | | | |  |  |
|  |  |  |  |  | Other (Specify) | | | | | | 5 | | |  | | | | | | | | |  |  |
| **Question No.** | **Section 1: Respondent’s Background** | | | **Response** | | | | | | | | | | | | | | | | | | | | **Code** |
| 101 | In what month and year were you born?  *Munabadwa mwezi uti, ndiponso chaka chanji?* | | | Month | | | | └─┴─┘  mm | | | | | | | | | | | | | | | |  |
|  |  |  |  | Don’t know month | | | | 98 | | | | | | | | | | | | | | | |  |
|  |  |  |  | Year | | | | └─┴─┴─┴─┘  year | | | | | | | | | | | | | | | |  |
|  |  |  |  | Don’t know year | | | | 9998 | | | | | | | | | | | | | | | |  |
| 102 | How old were you at your last birthday?  *Munakwanitsa zaka zingati pa tsiku lanu la kubadwa lapitalo?*  Compare and correct 101 and/or 102 if inconsistent | | | Age in complete years | | | | └─┴─┘  Years old | | | | | | | | | | | | | | | |  |
| 103 | Have you ever attended school?  *Kodi sukulu munayimbapo?* | | | Yes | | | | 1 | | | |  | | | | | | | | | | | |  |
|  |  |  |  | No | | | | 0 | | | | **If No, Go to Q.106** | | | | | | | | | | | |  |
| 104 | What is the highest level of school you attended: primary, secondary or higher?  *Kodi sukulu munafika nayo pati: pulaimale, sekondale kapena koleji?* | | | Primary | | | | 1 | | | |  | | | | | | | | | | | |  |
|  |  |  |  | Secondary | | | | 2 | | | |  | | | | | | | | | | | |  |
|  |  |  |  | Higher | | | | 3 | | | |  | | | | | | | | | | | |  |
| 105 | What is the highest level (grade/standard/form/year) you completed at that level?  *Kodi sukulu munasiyira kalasi iti (sitandadi, folumu, chaka)?*  If completed less than one year at that level record ‘00’ | | | Std/Form/Year  ***Tick where applicable here*** | | | | └─┴─┘ | | | | | | | | | | | | | | | |  |
| 106 | What is your religion?  *Kodi ndinu a mpingo wanji?* | | | Catholic | | | | 01 | | | | | | | | | | | | | | | |  |
|  |  |  |  | CCAP | | | | 02 | | | | | | | | | | | | | | | |  |
|  |  |  |  | Anglican | | | | 03 | | | | | | | | | | | | | | | |  |
|  |  |  |  | Seventh Day Advent/Bapti | | | | 04 | | | | | | | | | | | | | | | |  |
|  |  |  |  | Other Christian | | | | 05 | | | | | | | | | | | | | | | |  |
|  |  |  |  | Muslim | | | | 06 | | | | | | | | | | | | | | | |  |
|  |  |  |  | No Religion | | | | 07 | | | | | | | | | | | | | | | |  |
|  |  |  |  | Other (Specify) | | | | 96 | | | | | | | | | | | | | | | |  |
| 107 | What is your tribe or ethnic group?  *Kodi ndinu a mtundu wanji?* | | | Chewa | | | | 01 | | | | | | | | | | | | | | | |  |
|  |  |  |  | Tumbuka | | | | 02 | | | | | | | | | | | | | | | |  |
|  |  |  |  | Lomwe | | | | 03 | | | | | | | | | | | | | | | |  |
|  |  |  |  | Tonga | | | | 04 | | | | | | | | | | | | | | | |  |
|  |  |  |  | Yao | | | | 05 | | | | | | | | | | | | | | | |  |
|  |  |  |  | Sena | | | | 06 | | | | | | | | | | | | | | | |  |
|  |  |  |  | Nkhonde | | | | 07 | | | | | | | | | | | | | | | |  |
|  |  |  |  | Ngoni | | | | 08 | | | | | | | | | | | | | | | |  |
|  |  |  |  | Other (Specify) | | | | 96 | | | | | | | | | | | | | | | |  |
|  |  |  |  |  |  |  |  |  |  |  |  |  |  |  |  |  |  |  |  |  |  |  |  |  |
| 108 | What is your marital status?  *Kodi muli pa banja?* | | | Never married | | | | 01 | | | | | | | | | | | | | | | |  |
|  |  |  |  | Currently married | | | | 02 | | | | | | | | | | | | | | | |  |
|  |  |  |  | Separated | | | | 03 | | | | | | | | | | | | | | | |  |
|  |  |  |  | Divorced | | | | 04 | | | | | | | | | | | | | | | |  |
|  |  |  |  | Widowed | | | | 05 | | | | | | | | | | | | | | | |  |
|  |  |  |  | Cohabiting | | | | 06 | | | | | | | | | | | | | | | |  |
|  |  |  |  | Refused | | | | 98 | | | | | | | | | | | | | | | |  |
| 109 | Which of the following describes your main work status over the past 12 months?  *Kodi ntchito yanu yeniyeni ndi yotani pa miyezi 12 yapitayi?* | | | Government employee | | | | 01 | | | | | | | | | | | | | | | |  |
|  |  |  |  | Non-government employee | | | | 02 | | | | | | | | | | | | | | | |  |
|  |  |  |  | Self-employed | | | | 03 | | | | | | | | | | | | | | | |  |
|  |  |  |  | Non-paid | | | | 04 | | | | | | | | | | | | | | | |  |
|  |  |  |  | Student | | | | 05 | | | | | | | | | | | | | | | |  |
|  |  |  |  | Homemaker | | | | 06 | | | | | | | | | | | | | | | |  |
|  |  |  |  | Retired | | | | 07 | | | | | | | | | | | | | | | |  |
|  |  |  |  | Unemployed (able to work) | | | | 08 | | | | | | | | | | | | | | | |  |
|  |  |  |  | Unemployed (unable to work) | | | | 09 | | | | | | | | | | | | | | | |  |
|  |  |  |  | I don’t know/Refused | | | | 98 | | | | | | | | | | | | | | | |  |
| 110 | How many people older than 18 years, including yourself, live in your household?  *Kodi pakhomo panu pali anthu angati opyola / opitilira zaka 18 pamodzi ndi inu?* | | | └─┴─┘  Number of people | | | | | | | | | | | | | | | | | | | |  |
| 111 | Taking the past year, can you tell me what the average earnings of the household have been?  *Kodi munapeza ndalama zochuluka bwanji pa mulungu kapena pa mwezi kapena pa chaka mu chaka chapitachi?*  (Record only **One, Not** all 3) | | | └─┴─┴─┴──┴─┴─┴─┘  Per week | | | | | | | | | | | | | | | | | | | |  |
|  |  |  |  | OR  └─┴─┴─┴──┴─┴─┴─┘  Per month | | | | | | | | | | | | | | | | | | | |  |
|  |  |  |  | OR └─┴─┴─┴──┴─┴─┴─┘  Per year | | | | | | | | | | | | | | | | | | | |  |
|  |  | | |  | | | | | | | | | | | | | | | | | | | |  |
| 112 | Many different factors can prevent women from getting medical advice or treatment for themselves. When you are sick and want to get medical advice or treatment, is each of the following  big problem or not?  *Pali zifukwa zosiyanasiyana zomwe zimalepheretsa a mai apakati kupeza uphungu okhudzana ndi umoyo wao or chithandizo ku chipatala. Kumbali yanu, kodi mukadwa kapena mukufuna uphungu wa zamoyo, ndi zifukwa ziti zomwe zili zazikulu zimakubwezani mbuyo?* | | |  | | | | A big Problem  *Chifukwa chachikulu* | | | | | | | | Not a big Problem  *Chifukwa chaching’ono* | | | | | | | |  |
|  |  |  |  | Getting permission to go?  *Kupeza chilorezo kuchoka abambo?* | | | | 1 | | | | | | | | 2 | | | | | | | |  |
|  |  |  |  | Getting money needed for treatment?  *Ndalama zolipirila ku chipatala?* | | | | 1 | | | | | | | | 2 | | | | | | | |  |
|  |  |  |  | The distance to the health facility?  *Kutalika kwa mtunda waku chipatala?* | | | | 1 | | | | | | | | 2 | | | | | | | |  |
|  |  |  |  | Having to take transport?  *Mayendedwe?* | | | | 1 | | | | | | | | 2 | | | | | | | |  |
|  |  |  |  | Not wanting to go alone?  *Kusafuna basi?* | | | | 1 | | | | | | | | 2 | | | | | | | |  |
|  |  |  |  | Concern that there may not be a female health provider?  *Ganizo loti sindikapezako a dokotala achizimai?* | | | | 1 | | | | | | | | 2 | | | | | | | |  |
|  |  |  |  | Concern that there may not be any health provider?  *Ganizo loti sindikapeza ondipatsa thandizo?* | | | | 1 | | | | | | | | 2 | | | | | | | |  |
|  |  |  |  | Concern that there may be no drugs available?  *Ganizo loti ndikapeza kopanda mankhwala?* | | | | 1 | | | | | | | | 2 | | | | | | | |  |
| **Question No.** | **Section 2: Reproduction** | | | | | | **Response** | | | | | | | | | | | | | | | | | **Code** |
| 201 | Was this your first pregnancy?  *Iyi inali mimba yanu yoyamba?* | | | | | | Yes | | | 1 | | | **If Yes, Go to Q.203** | | | | | | | | | | |  |
|  |  |  |  |  |  |  | No | | | 0 | | | | | | | | | | | | | |  |
| 202 | How many pregnancies have u had during your life?  Kodi mwachembezako kangati? | | | | | | └─┴─┘ | | | | | | | | | | | | | | | | |  |
|  |  |  |  |  |  |  | Number of pregnancies | | | | | | | | | | | | | | | | |  |
|  |  | | | | | |  | | | | | | | | | | | | | | | | |  |
| 203 | Have you ever given birth?  *Munayamba mwaberekako?* | | | | | | Yes | | | 1 | | | | | | | | | | | | | |  |
|  |  |  |  |  |  |  | No | | | 0 | | | **If No, Go to Q.204** | | | | | | | | | | |  |
| 204 | How many births have you given during your life?  *Mwabelekako ana angati pa moyo wanu?* | | | | | | └─┴─┘ | | | | | | | | | | | | | | | | |  |
|  |  |  |  |  |  |  | Number of birth | | | | | | | | | | | | | | | | |  |
| **Question No.** | **Section 3: Pregnancy, Malaria and Intermittent Preventative Treatment** | | | | | **Response** | | | | | | | | | | | | | | | | | | **Code** |
| 301 | When you were pregnant with this new born baby, did you see anyone for antenatal care for this pregnancy?  *Pamene munali oyembekezera, mudabwerako kusikelo?* | | | | | Yes | | | | | | | | | 1 | | | | | | | | |  |
|  |  |  |  |  |  | No | | | | | | | | | 0 | | **If No, Go to Q.305** | | | | | | |  |
| 302 | Whom did you see?  *Ndi ndani amene anakupatsani chithandizo?* | | | | | **Health Personnel** | | | | | | | | |  | | | | | | | | |  |
|  |  |  |  |  |  | Doctor/Clinical Officer | | | | | | | | | A | | | | | | | | |  |
|  |  |  |  |  |  | Nurse/Midwif | | | | | | | | | B | | | | | | | | |  |
|  |  |  |  |  |  | Patient Attendant | | | | | | | | | C | | | | | | | | |  |
|  |  |  |  |  |  | HSA | | | | | | | | | D | | | | | | | | |  |
|  |  |  |  |  |  | **Other person** | | | | | | | | |  | | | | | | | | |  |
|  |  |  |  |  |  | Traditional Birth Attendant | | | | | | | | |  | | | | | | | | |  |
|  |  |  |  |  |  | Other (Specify) | | | | | | | | |  | | | | | | | | |  |
|  |  |  |  |  |  |  |  |  |  |  |  |  |  |  |  |  |  |  |  |  |  |  |  |  |
| 303 | How many times did you receive antenatal care during this pregnancy?  *Pa nthawi yomwe munali oyembekezera, mudapitako kangati ku sikelo?*  **CROSS-CHECK WITH ANC CARD** | | | | | └─┴─┘ | | | | | | | | | | | | | | | | | |  |
|  |  |  |  |  |  | Number of ANC visits | | | | | | | | | | | | | | | | | |  |
|  |  |  |  |  |  | Don’t know | | | | | | | | | 98 | | | | | | | | |  |
| 304 | How old was the pregnancy (in months) when you first sought/visited antenatal care?  *Kodi pamene munkayamba sikelo, munali ndi pakati pa miyezi ingati?* | | | | | └─┴─┘ | | | | | | | | | | | | | | | | | |  |
|  |  |  |  |  |  | Number of months (Gestation period) | | | | | | | | | | | | | | | | | |  |
|  |  |  |  |  |  | Don’t know | | | | | | | | | 98 | | | | | | | | |  |
| 305 | How is malaria transmitted?  *Mwanjira izi, sankhani njira zomwe mukudziwa kuti malungo amafalila?* | | | | |  | | | | | | | | | Yes | | | | | No | | | |  |
|  |  |  |  |  |  | Eating un matured sugar cane  *Kudya nzimbe zosankhwima* | | | | | | | | | 1 | | | | | 0 | | | |  |
|  |  |  |  |  |  | Mosquito *Udzudzu* | | | | | | | | | 1 | | | | | 0 | | | |  |
|  |  |  |  |  |  | Witchcraft *Ufiti* | | | | | | | | | 1 | | | | | 0 | | | |  |
|  |  |  |  |  |  | Don’t know | | | | | | | | | 1 | | | | | 0 | | | |  |
| 306 | What are the dangers of malaria in pregnancy?  *Kodi malungo amabweretsa mavuto wotani kwa amai apakati?* | | | | |  | | | | | | | | | Yes | | | | | No | | | |  |
|  |  |  |  |  |  | Abortion *Pathupi kuchoka* | | | | | | | | | 1 | | | | | 0 | | | |  |
|  |  |  |  |  |  | Still birth *kupititsa padela* | | | | | | | | | 1 | | | | | 0 | | | |  |
|  |  |  |  |  |  | Low birth weight  *Mwana kubadwa onyetchera?* | | | | | | | | | 1 | | | | | 0 | | | |  |
| 307 | Have you ever heard or informed about malaria prevention during pregnancy using SP/Fansidar?  *Kodi munavapo za mankhwala a fansidar ngati njira imodzi yoteteza amai apakati ku malungo?* | | | | | Yes | | | | | | | | | 1 | | | | | | | | |  |
|  |  |  |  |  |  | No | | | | | | | | | 0 | | | | | | | | |  |
| 308 | During this pregnancy, did you take SP/Fansidar or Novidar SP to keep you from getting malaria?  *Kodi munamwako mankhwala a fansidar panthawi yomwe munali woyembekezera?* | | | | | Yes | | | | | | | | | 1 | | | | | | | | |  |
|  |  |  |  |  |  | No | | | | | | | | | 0 | | | | | | **If No or don’t know, Go to S.4** | | |  |
|  |  |  |  |  |  | Don’t know | | | | | | | | | 98 | | | | | |  |  |  |  |
| 309 | How many times did you take SP/ Fansidar or Novidar SP during this pregnancy?  *Ndimaulendo angati omwe munamwa fansidar?* | | | | | └─┴─┘ | | | | | | | | | | | | | | | | | |  |
|  |  |  |  |  |  | Times | | | | | | | | | | | | | | | | | |  |
| 310 | Where did you get SP/ Fansidar or Novidar SP from?  *Mankhwala amenewa munawalandilila kuti?* | | | | | Antenatal visit | | | | | | | | | 1 | | | | | | | | |  |
|  |  |  |  |  |  | Another facility visit | | | | | | | | | 2 | | | **If 2 or 3, Go to S.4** | | | | | |  |
|  |  |  |  |  |  | Other sources-specify: | | | | | | | | | 3 | | |  |  |  |  |  |  |  |
| 311 | How many times did you take SP/ Fansidar or Novidar SP during an antenatal visit?  *Ndimaulendo angati amene munalandira fansidar mutapita ku sikelo?* | | | | | └─┴─┘ | | | | | | | | | | | | | | | | | |  |
|  |  |  |  |  |  | Times | | | | | | | | | | | | | | | | | |  |
| 312 | Did you take SP/ Fansidar or Novidar SP under direct observation by the health worker each time?  *Kodi a dokotala amakhala ali pompo kuonetsetsa kuti mwamwa fansidar pa nthawi zonse zomwe mumalandila mankhwalawa ku sikelo?* | | | | | Yes | | | | | | | | | 1 | | | | **If Yes, Go to S.4** | | | | |  |
|  |  |  |  |  |  | No | | | | | | | | | 0 | | | | | | | | |  |
| 313 | How many times did you take SP under observation by health worker?  *Ndimaulendo angati omwe munamwa Fansidar pamaso pa dokotola?* | | | | | └─┴─┘ | | | | | | | | | | | | | | | | | |  |
|  |  |  |  |  |  | Times | | | | | | | | | | | | | | | | | |  |

| **Question No.** | **Section 4: Iron supplements during pregnancy** | **Response** | | | | **Code** |
| --- | --- | --- | --- | --- | --- | --- |
| 401 | During this pregnancy, were you given or did you buy any iron tablets?  *Kodi munamwako mankhwala owonjezela magazi panthawi yomwe munali woyembekezera?*  SHOW TABLETS | Yes | 1 | | |  |
|  |  | No | 0 | | **If No or don’t know, Go to S.5** |  |
|  |  | Don’t know | 98 | |  |  |
| 402 | During the whole pregnancy, for how many days did you not take the tablets?  *Panthawi yonse munali oyembekezera, kodi ndimasiku angati omwe simunamwa mankhwala owonjezela magaziwa?*  IF ANSWER IS NOT NUMERIC, PROBE FOR APPROXIMATE NUMBER OF DAYS | └─┴─┴─┘ | | | |  |
|  |  | Days | | | |  |
|  |  | Don’t know | | 98 | |  |
|  |  |  |  |  |  |  |

| **Question No.** | **Section 5: Alcohol use and tobacco smoking** | **Response** | | **Code** |
| --- | --- | --- | --- | --- |
| 501 | How often during the past **nine** **months** did you have a drink containing alcohol?  *Kodi ndikangati komwe munamwako chakumwa choledzeletsa pa mwezi isano ndi inayi yapitayi?* | Never *Sindinamweko* | 0 |  |
|  |  | Monthly or less  *Kamodzi pa mwezi* | 1 |  |
|  |  | 2-4 times a month  *Kawiri, katatu ndi kanai pa mwezi* | 2 |  |
|  |  | 2-3 times a week  *Kawiri kapena katatu pa mulungu* | 3 |  |
|  |  | 4 or more times a week  *Kanai or kupyolera apo pa mulungu* | 4 |  |
| 502 | Have you ever smoked tobacco cigarettes?  *Kodi munasutapo fodya?* | Never smoked | 0 |  |
|  |  | Yes, but not in the past **nine** **months** | 1 |  |
|  |  | Yes, in the past **nine** **months** | 2 |  |
|  |  | Prefer not to answer | 99 |  |

| **Question No.** | **Section 6: Woman’s maternal health** | | **Response** | | | | | | | | | **Code** |  |
| --- | --- | --- | --- | --- | --- | --- | --- | --- | --- | --- | --- | --- | --- |
|  | **History of Raised blood pressure** | |  | |  | |  | | | | |  |  |
| 601 | During the past **nine months** have you been told by a doctor or health worker that you have raised blood pressure or hypertension?  *Kodi pa miyezi 9 yapitayi a dokotala anakupimaniko ndikukupezani ndi matenda othamanga magazi (BP)?* | | No | | 0 | |  | | | | |  |  |
|  |  |  | Yes | | 1 | | **CROSS-CHECK WITH ANC CARD** | | | | |  |  |
|  | **History of anaemia** | |  | |  | |  | | | | |  |  |
| 602 | During the past **nine months** have you been told by a doctor or other health worker that you have anaemia?  *Kodi pa miyezi 9 yapitayi a dokotala anakupimaniko ndikukupezani ndi matenda ochepa magazi?* | | No | | 0 | |  | | | | |  |  |
|  |  |  | Yes | | 1 | | **CROSS-CHECK WITH ANC CARD** | | | | |  |  |
|  | **History of diabetes** | |  | |  | |  | | | | |  |  |
| 603 | During the past **nine months** have you been told by a doctor or other health worker that you have diabetes?  *Kodi pa miyezi 9 yapitayi a dokotala anakupimaniko ndikukupezani ndi matenda a suga?* | | No | | 0 | |  | | | | |  |  |
|  |  |  | Yes | | 1 | | **CROSS-CHECK WITH ANC CARD** | | | | |  |  |
| 604 | During the whole pregnancy, have you ever got sick with the following illness?  *Kodi anakupezanipo ndi matenda awa munyengo yomwe munali ndipakati?* | |  | | | | | | Yes | No | |  |  |
|  |  |  | Urinary tract infection  *Matenda achikhodzodzo* | | | | | | 1 | 0 | |  |  |
|  |  |  | Pneumonia *chibayo* | | | | | | 1 | 0 | |  |  |
|  |  |  | STIs *Matenda opatsirana pakugonana* | | | | | | 1 | 0 | |  |  |
|  |  |  | Hepatitis *Matenda achiwindi* | | | | | | 1 | 0 | |  |  |
|  |  |  | Other (Specify) | | | | | |  | | |  |  |
|  |  |  |  |  |  |  |  |  |  |  |  |  |  |
| **Question No.** | | **Section 7: Physical Measurements** | | **Response** | | | | | | | **Code** | | |
| 701 | | Was your newly born baby weighed at birth?  *Kodi mwana wanu anamupima sikelo atangobadwa?* | | Yes | | 1 | | | | |  | | |
|  |  |  |  | No | | 0 | | **If No or don’t know, Go to Q.703** | | |  |  |  |
|  |  |  |  | Don’t know | | 98 | |  |  |  |  |  |  |
|  | |  | |  | |  | |  | | |  | | |
| 702 | | How much did she/he weigh?  *Kodi sikelo yake imalemela bwanji?*  RECORD WEIGHT IN KILOGRAMS FROM MOTHER’S HEALTH CARD, IF AVAILABLE | | **a. Kg from Card** | | └─┴─┴─┘. └─┘ | | | | |  | | |
|  |  |  |  |  |  | Kilograms | | | | |  |  |  |
|  |  |  |  | **b. Kg from Recall** | | └─┴─┴─┘. └─┘ | | | | |  |  |  |
|  |  |  |  |  |  | Kilograms | | | | |  |  |  |
|  |  |  |  | Don’t know | | 9998 | | | | |  |  |  |
| 703 | | What was your weight from the first antenatal care visit measurement?  *Kodi mene mumadzayamba sikelo, mumalemera bwanji?*  RECORD WEIGHT IN KILOGRAMS FROM MOTHER’S HEALTH CARD, IF AVAILABLE | | **a. Kg from Card** | | └─┴─┴─┘. └─┘ | | | | | **If measurement known, Go to Q.705** | | |
|  |  |  |  |  |  | Kilograms | | | | |  |  |  |
|  |  |  |  | **b. Kg from Recall** | | └─┴─┴─┘. └─┘ | | | | |  |  |  |
|  |  |  |  |  |  | Kilograms | | | | |  |  |  |
|  |  |  |  | Don’t know | | 9998 | | | | |  | | |
| 704 | | Weight from the current measurement.  TAKE WEIGHT OF THE PARTICIPANT | | └─┴─┴─┘. └─┘ | | | | | | |  | | |
|  |  |  |  | Kilograms | | | | | | |  |  |  |
| 705 | | What was your height from the first antenatal care visit measurement?  *Nanga kodi mulingo wakutalika kwanu unali otani panthawi yomwe mumadzayamba sikelo?* | | **a. Height from Card** | | └─┴─┴─┘. └─┘ | | | | | **If measurement known, Go to Q.707** | | |
|  |  |  |  |  |  | Centimetres | | | | |  |  |  |
|  |  |  |  | **b. Height from Recall** | | └─┴─┴─┘. └─┘ | | | | |  |  |  |
|  |  |  |  |  |  | Centimetres | | | | |  |  |  |
|  |  |  |  | Don’t know | | 9998 | | | | |  | | |
| 706 | | Height from the current measurement.  TAKE HEIGHT OF THE PARTICIPANT | | └─┴─┴─┘. └─┘ | | | | | | |  | | |
|  |  |  |  | Centimetres | | | | | | |  |  |  |
| 707 | | Body Mass Index (BMI)  FROM Q. 503 AND Q. 505 OR Q.504 AND Q.506 CALCULATE BMI =Weight (in kilogram)÷ height squared (in centimeters) | | └─┴─┘. └─┴─┘ | | | | | | |  | | |
|  |  |  |  | BMI (kg/cm^2^) | | | | | | |  |  |  |

*Most questions adapted from Malawi Malaria Survey Indicator 2015 and 2010 Malawi DHS

**Mafunso athera pamenepa! Zikomo kwambiri polora kuti tikufunseni mafunso**
